# The Cross-Bridge of Skeletal Muscle Is Not Synchronized Either by Length or Force Step

**DOI:** 10.3390/ijms160612064

**Published:** 2015-05-27

**Authors:** Enrico Grazi

**Affiliations:** Department of Scienze Biomediche e Chirurgiche Specialistiche, Ferrara University, Via Borsari 46, 44121 Ferrara, Italy; E-Mail: enrico.grazi@unife.it; Tel.: +39-532-248642; Fax: +39-532-294031

**Keywords:** skeletal muscle contraction, cross-bridges synchronization, interference distance, power stroke

## Abstract

Force and length steps, applied to a muscle fiber in the isometric state, are believed to synchronize attached cross-bridges. This alleged synchronization facilitates the interpretation of the experiments. A rapid force step elicits an elastic response of the attached cross-bridges, followed by an isotonic phase. The decay of this second isotonic phase is of the first order. This excludes that the attached cross-bridges may decay all at the same time. The change of the X-ray interference distance during the second phase measures the stroke size only in the unrealistic case that the cross-bridges are and remain all attached. A rapid force step does not synchronize attached cross-bridges. The change of X-ray interference during the second phase does not measure the stroke size. These conclusions significantly change the picture of the mechanism of skeletal muscle contraction.

## 1. Introduction

The concept that either force or length steps, applied to a muscle fiber in the isometric state, synchronize attached cross-bridges was introduced by Huxley and Simmons [1]. This view, although not really supported by experimental evidence, is still very popular, probably because it helps in the interpretation of the experiments.

By now many authors [2,3,4,5,6,7,8,9,10] addressed this topic and, to validate their conclusion, it is important to establish whether or not force and length steps are indeed able to synchronize attached cross-bridges.

Here experimental evidences are presented that disprove the claim of Huxley and Simmons [1]. I show that, in the course of either force or length steps, the decay of the attached cross-bridges is of the first order, thus it cannot be simultaneous. I also show that the lack of a simultaneous decay nullifies the attempts to deduce the myosin stroke size from the interference of the 14.57 nm meridional X-ray reflection. Therefore, the conclusions made on the assumption that attached cross-bridges decay simultaneously must be revised.

The work of Piazzesi *et al.* [2] and of Reconditi *et al.* [3] is particularly suitable to examine the above aspects.

**Figure 1:**
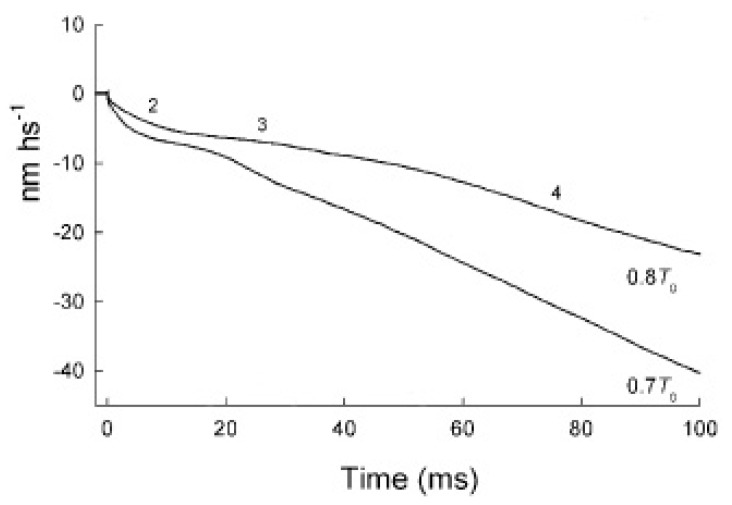
Length responses following a step change in force. The numbers indicate the phases. Adapted from figure 1B of [2].

Piazzesi *et al.* [2], with 150 µs force steps, separated the elastic response of the attached cross-bridges and filaments (phase 1) from the subsequent isotonic phases of the velocity transient. Of these, the rapid phase 2 was claimed to represent the synchronous execution of the working stroke. The slower phases 3 and 4 were assigned to detachment and attachment of the myosin head with the subsequent filament sliding (Figure 1). Piazzesi *et al.* [2] showed that the speed and the amplitude of phase 2 increases with the amplitude of the force step. They concluded that the step size of the power stroke increases from 4 nm at 0.8 *T*_0_ to ~7 nm at *T* = 0 (where *T*_0_ is the isometric tension and *T* is the actual tension of the fiber).

Reconditi *et al.* [3], by studying the interference of the 14.57 nm meridional X-ray reflection (M3), attempted to determine the myosin stroke size. By applying a rapid force step to the muscle fiber, they expected to generate a synchronous motion of the attached myosin heads and to alter the axial position of the center of scattering mass by a calculable amount. From the shift of the position of the center of the scattering mass Reconditi *et al.* [3] presumed to calculate the stroke size.

## 2. Results

### 2.1. The Rapid Phase 2 Cannot Be Related to the Synchronous Execution of the Working Stroke

**Figure 2:**
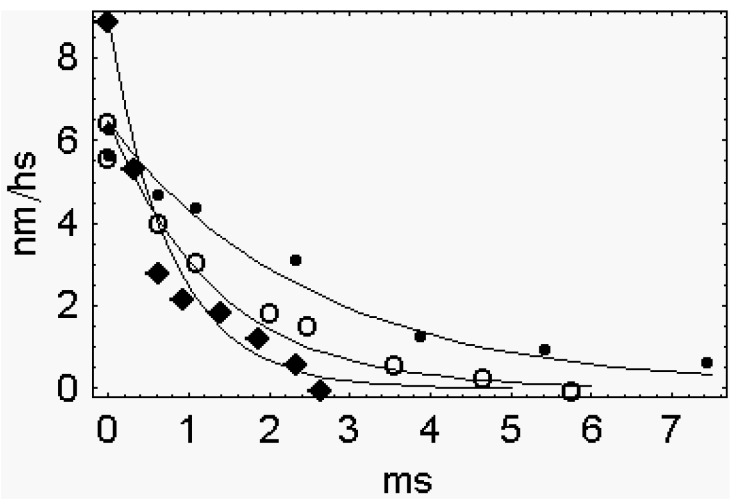
The decay of phase 2 as a first order phenomenon. Data are taken from figure 1B of Piazzesi *et al.* [2]. For drawing the graph the length zero of figure 1B is used as a common origin. The origin of the different traces is obtained by subtracting, from the common origin, the shortening simultaneous with the force step, L_1_ (Figure 2A of Piazzesi *et al.* [2]. Filled diamond, 0.3 *T*_0_; open circle, 0.7 *T*_0_; filled circle, 0.8 *T*_0_. The continuous lines are obtained by the equation, l = l_0_ Exp[−k t].

The hypothesis that the rapid phase 2 is related to the synchronous execution of the working stroke is disproved by the data themselves of Piazzesi *et al.* [2]. Their figure 1B shows that phase 2 decays with the apparent first order rate constants of 1300 s^−1^ at 0.3 *T*_0_, of 1400 s^−1^ at 0.5 *T*_0_, of 750 s^−1^ at 0.7 *T*_0_ and of 400 s^−1^ at 0.8 *T*_0_ (Figure 2).

A reaction of the first order implies that the decay occurs randomly and not at all as a single event, as it should be in the case of the simultaneous power stroke of the synchronized cross-bridges. A single event means that phase 2 should show up as a step and not as a first order reaction. It is concluded that the difference between the elastic response (L_1_) and total shortening at the end of phase 2 (L_2_) does not represent the step size of the power stroke. It represents, on the contrary, the summation of the steps performed in a stochastic sequence by many cross-bridges.

An attempt to provide an order of magnitude to the step size of the power stroke can be made by considering that there are 294 cross-bridges on half myosin filament [11] and that, in the isometric state, only 94 (32%) are attached [12].

Furthermore, because of the 1:2 thick to thin filament stoichiometry, the 94 cross-bridges should be shared, 47 to 47, between two thin filaments. If the number of the attached cross-bridges decreases proportionally to the tension, then, as the average, the number of attached cross-bridges per thin filament should be 37–38 at 0.8 *T*_0_ and 4–5 at 0.1 *T*_0_. It is concluded that, at 0.8 *T*_0_, 37–38 cross-bridges contribute to phase 2. As a consequence the length step of the power stroke is not ~4 nm but, at best, ~4/37 = 0.108 nm. At 0.1 *T*_0_, ~5 cross-bridges contribute to phase 2. As a consequence, the length step of the power stroke is not ~7 nm but, at best, ~7/5 = ~1.4 nm.

### 2.2. In Phase 2 the Change of the Interference Distance Is Not Solely Related to the Power Stroke

Each half-thick filament consists of 49 layers of heads [11,13] with axial periodicity 14.573 nm during active contraction. The two head arrays are separated by a bare zone of approximately 160 nm in the center of the filament. Thus, the geometrical centers of the two arrays are separated by 48 times the axial periodicity plus the bare zone: 14.573 × 48 + 160 = 859.504 nm [13,14].

The recording, at very high spatial resolution, of the 14.57 nm meridional reflection (M3) shows that the reflection consists of two close-spaced peaks of unequal intensity [15]. The splitting is caused by interference effects between the diffractions from the arrays of cross-bridges in the two halves of each thick filament [16].

From the fine structure of the M3 reflection, *i.e.*, the intensity ratio and the spacing of the peaks, the interference distance (ID), *i.e.*, the distance between the centers of mass of the two arrays, can be calculated. Thus a change of the interference distance signals a displacement of the position of the center of mass of the two arrays. The position of the center of mass does not always coincide with the geometrical center since it is a function of both the tilting of the heads and of the distribution of the attached and of the detached heads.

The position of the centers of mass of the two arrays could be calculated provided that the distribution of the attached and of the detached heads and the dispersion of their lever arms were known. We present here two simple models.

#### 2.2.1. The First Model

We compare two cases. In the first case all the cross-bridges are attached and display the same angle of tilt. The angles of tilt are either 90° or 70° or 50° with respect to the filament axis.

With the tilt at 90°, the centers of mass coincide with the geometric centers and the interference distance is 859.504 nm. With the tilt at 70°, the interference distance becomes 853.86 nm. The difference between the interference distances at 90° and 70° equals exactly the projection of the cross-bridge on the filament axis, *i.e.*, the step size of the power stroke:

859.504 − 853.86 = 16.5 Cos[70°] = 5.64 nm
(1)

where, 16.5 nm, is the length of myosin subfragment-1 [17].

With the tilt at 50° the interference distance is 848.898 nm. The difference between the interference distances at 90° and 50° equals exactly the projection of the cross-bridge on the filament axis, *i.e.*, the step size of the power stroke:

859.504 − 848.898 = 16.5 Cos[50°] = 10.606 nm
(2)

In the second case the attached and the detached cross-bridges are alternating. The angles of tilting are either 90° for the attached cross-bridges and 60° for the detached, or 70° and 60°, or 50° and 60°, with respect to the filament axis (Appendix A).

In no case the difference between the interference distances coincides with the step size of the power stroke (Table 1).

This simple model shows that, only if all the cross-bridges are attached and display the same angle of tilt, the difference between the interference distances of two states measures the step size of the power stroke. It is sufficient to mix up the pattern by adding detached cross-bridges, albeit with the same angle of tilt, to break down the procedure. In muscle fiber the situation is even worst because of the spreading of the tilt angles.

**Table 1:** Length of the hypothetical power stroke and of the change of the interference distance (ΔID) in the presence and in the absence of detached cross-bridges. Hypothetical stroke = 16.5 Cos[α], where, α, is the tilt angle of the attached cross-bridges. ΔID is the difference between the interference distances at 90° and either at 70° or at 50°.

Attached, Tilt Angle	Detached, Tilt Angle	Hypothetical Stroke (nm)	ΔID (nm)
70°	none	5.643	5.643
50°	none	10.606	10.606
70°	60°	5.643	2.764
50°	60°	10.606	5.194

#### 2.2.2. The Second Model

##### The Attached Head Domains

Let assume that, in the second phase, attached cross-bridges tilt from 140° to 104°, with reference to the filament axis.

The myosin subfragment-1 is 16.5 nm long [17] and is composed by a part of 6.7 nm, fixed to actin (relative mass 0.4), and a rotating part of 9.8 nm (relative mass 0.6) [18].

Thus the step size of the power stroke is:

9.8 (Cos[140°] − Cos[104°]) = −5.1364 nm
(3)

Under these conditions, provided that the arrays are fully occupied by attached cross-bridges whose rotating part tilt from 140° to 104°, the difference between the interference distance at the beginning and at the end of the second phase equals the stroke size.

##### The Detached Head Domains

The detached head domains are assumed to be in the extended configuration, 16.5 nm. Some of them share the head-rod junction with an attached head domain, the others share the head-rod junction with another detached head domain. In the program the higher limit of the attached cross-bridges is set to 32% and the angle of tilt of the detached head domains is selected randomly (Appendix B).

**Figure 3:**
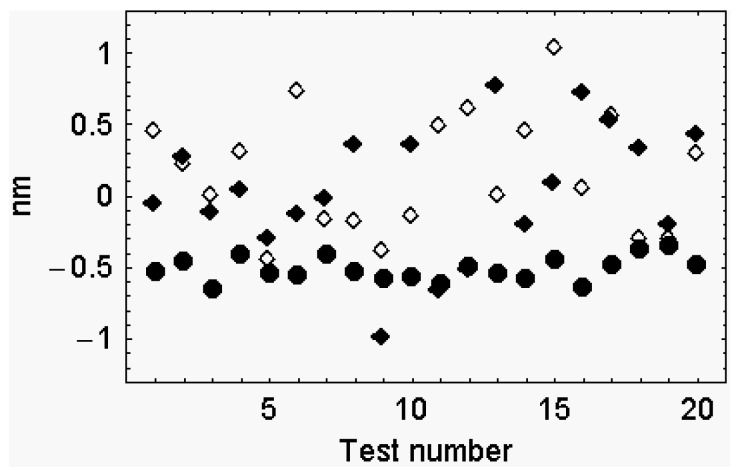
The difference between the sums of the positions of the centers of mass of the myosin arrays, at the beginning and at the end of phase 2, as a function of the tilt angles of the detached head domains. The upper limit of the attached head domains was set to 32%. The average angle of tilt was 140° at the beginning of the second phase and 104° at the end of the second phase. The angle of tilt of the detached head domains was selected at random between: **1** detached coupled with detached head domain, 150°–70°; detached coupled with attached head domain, 120°–90° (empty diamond): **2** all the detached head domains tilt between 150° and 40° (filled diamond); **3** all the detached head domains are at 90° (filled circle). The size of the working stroke is at −5.1364 nm.

The proposition of Reconditi *et al.* [3], that the difference between the interference distances at the beginning and at the end of phase 2 equals the stroke size, is verified only if the cross-bridges are all attached and with the same angle of tilt. On the contrary, the proposition of Reconditi *et al.* [3] fails when the fraction of the attached cross-bridges is below 100%, even though the angle of tilt of the detached cross-bridges is set to 90°, to neutralize their influence on the position of the center of mass of the myosin arrays. Even more the proposition of Reconditi *et al.* [3] fails when the attached cross-bridges are below 100% and the angles of tilt of detached cross-bridges are selected at random (Figure 3).

## 3. Discussion

I did already question the hypothesis that a length step may synchronize attached cross-bridges [19]. This is because, during contraction, cross-bridges are functionally asynchronous [20]. Moreover, during quick releases, the dispersion of the cross-bridges remains approximately constant. Thus the mean distribution of the cross-bridges is not altered by the quick release even though the tension developed decreases drastically [18,21]. It seems therefore safe to conclude that quick release does not improve the “synchronization” of the cross-bridges. To the remarks of H.E. Huxley [20] I add now new and more stringent reasons.

In the work of Piazzesi *et al.* [2] the decay of phase 2 is of the first order. This modality precludes the possibility that the attached cross-bridges decay all at the same time. They must decay at different times. The whole phase 2 arises from the summation of the separate length decays of the attached cross-bridges. For this reason, the length decay of phase 2 cannot represent the step size of the working stroke.

It is clear that “stepping of all motors at the same time” and the “individual motors, operating stochastically in sequence” do not display the same effect. In the second case the system is perturbed sequentially by each individual motor. “Macroscopically” the regime is isotonic but, “microscopically”, tension is continuously restored by the measuring apparatus [22]. The system being synchronous or asynchronous is not without effect on modeling. According to Caremani *et al.* [10] the synchronized termination of the working stroke causes instability, due to a transient deficit of the force generation. To eliminate this instability Caremani *et al.* [10] propose that motors may slip on the next actin monomer thus generating supplementary work to execute phase 3. The question is: what happens to the slipping hypothesis if cross-bridge synchronization does not take place?

More generally, the theoretical formalisms of Huxley and Simmons [1] and of TL Hill [23] are probably not adequate to describe muscle contraction. Mechanical models must be integrated with the notion that muscle is a highly non-ideal system [24,25].

Reconditi *et al.* [3], from the axial X-ray intensity distribution in the region of the M3 reflection, calculate the difference between the interference distances at the beginning and at the end of phase 2. According to them this difference equals the stroke size. The interference distance is the distance between the centers of mass of the two arrays of the thick filament. Thus the interference distance could be calculated provided that the positions of the centers of mass were known.

I made two simple models. 1. In the first model two situations are confronted: (a) the cross-bridges are all attached and have the same angle of tilt; (b) attached and detached cross-bridges alternate: attached cross-bridges display the angle of tilt α, detached cross-bridges display the angle of tilt β. Only in the first case the difference between the interference distances equals the stroke size.

In the second model the attached cross-bridges display all the same angle of tilt and their upper limit is set to 0.32%. The tilt angles of the detached cross-bridges are selected at random. The radius of rotation of the attached cross-bridges is 9.8 nm and that of the detached cross-bridges is 16.5 nm. Also in this case the changes of the interference distance do not provide information on the stroke size unless the myosin arrays are populated exclusively by attached cross-bridges with identical tilting angles. The model of Reconditi *et al.* [3] was confuted also by Knupp *et al.* [26]. According to these authors X-ray interference provides little direct information about the position of the myosin head lever arm. Knupp *et al.* [26] further observe that, beside the interference across the A-band, the meridional M3 X-ray intensity changes can all be explained by the changing diffraction effects during filament sliding caused by heads attached to actin moving axially relative to a population of detached heads that remain fixed in position relative to the myosin filament backbone. To Knupp *et al.* [26] respond Fusi *et al.* [27] maintaining to have always taken into account the influence of the detached heads.

## 4. Experimental Section

### 4.1. Cross-Bridge Synchronization

Kinetic analysis was used to ascertain whether or not cross-bridges are synchronized by force steps. A first order decay of phase 2 would exclude synchronization.

### 4.2. The Center of Mass of an Array of Cross-Bridges

The position of the center of mass of a linearly ordered array of layers is given by:

(m_1_ x_1_ + m_2_ x_2_ + m_3_ x_3_ +…. + m_n-1_ x_n-1_ + m_n_ x_n_)/(m_1_ + m_2_ + m_3_ +….+ m_n-1_ + m_n_) (4)

where, the m, indicate the masses of the arrays and, x, the coordinate of the linearly ordered arrays.

Since in our case the masses of the clusters are equal the formula simplifies to:

(x_1_ + x_2_ + x_3_ +…. + x_n-1_ + x_n_)/n (5)

## 5. Conclusions

The first order kinetics of phase 2 precludes the possibility that attached cross-bridges decay simultaneously. This means that attached cross-bridges are not synchronized.

The X-ray interference of the 14.5 nm meridional reflection (M3) can measure the size of the stroke only if cross-bridges are all attached and display the same angle of tilt.

## Appendix A

This model of the thick filament is a simplified one. The half-thick filament consists of 49 layers of heads [4,6], but each layer is equipped with only one cross-bridge. During the active contraction the axial periodicity of the layers is d = 14.573 nm. The length of the array is la = 48 × d = 699.504 nm. The bare zone, bz, is 160 nm long. The distance between the geometric centers of the two arrays is, 2 × 349.752 + 160 = 859.504 nm.

The position of the center of mass of a linearly ordered array of layers is given by:

(m_1_ x_1_ + m_2_ x_2_ + m_3_ x_3_ +…. + m_n-1_ x_n-1_ + m_n_ x_n_)/(m_1_ + m_2_ + m_3_ +….+ m_n-1_ + m_n_)
(6)

where, the m, indicate the masses of the arrays and, x, the coordinate of the linearly ordered arrays.

Since in our case the masses of the clusters are equal the formula simplifies to:

(x_1_ + x_2_ + x_3_ +…. + x_n-1_ + x_n_)/n
(7)

In our model the position of the center of mass of any myosin head, c_M_, is:

c_M_ = 16.5 Cos[α]/2 (nm)
(8)

where, α, is the tilting angle and 16.5 nm is the length of the myosin head [10].

The position of the center of mass of a myosin head in a particular layer, c_ML_, is:

c_ML_ = la + c_M_ (nm)
(9)

where, la = n × d, with, n, from 0 to 48, is the axial coordinate of the S1-S2 junction in any particular layer.

The position of the center of mass of the array, c_AR_, is:

c_AR_ = 48 × d − Sum[c_ML_, {n, 0, 48}]/49
(10)

Once the center of mass of the array is known the interference distance, ID, is calculated:

ID = 2 × c_AR_ + bz
(11)

The whole program is:

d = 14.573;

aa = Table[{n,

la = n d;

If[OddQ[n],cm = 16.5 Cos[70 Degree]/2, cm = 16.5 Cos[60 Degree]/2];

cml = la + cm; la, cm, cml},{n,0,48}];

car = 48 d − N[Apply[Plus, aa[[Range[49],4]]]/49]

The line below allows the choice between two alternatives:

If[OddQ[n],cm = 16.5 Cos[70 Degree]/2, cm = 16.5 Cos[60 Degree]/2];

If n is an odd number, 16.5 Cos[70 Degree]/2, is selected. If, n, is an even number, 16.5 Cos[60 Degree]/2, is selected.

When all the cross-bridges are attached, both the choices are filled with the same expression.

## Appendix B

In this model each crone is composed by three couples of cross-bridges. Each couple is composed either by an attached plus a detached head domain or by two detached head domains. The angle of tilt of the attached heads is fixed and their fraction is limited to 0.32. The angle of tilt of the detached head domains is selected at random.

The detached head domains are in the extended configuration, 16.5 nm. Their mass is unitary so it elides. The attached head domains are composed by a part of 6.7 nm, fixed to actin, relative mass = 0.4; and by a rotating part of 9.8 nm, relative mass = 0.6 [18]. The position of their center of mass is:

m_1_ or + m_2_ (or + 9.8 Cos[α]/2)
(12)

where, or, is the position of the origin of the crone and, α, is the angle of tilt of the attached cross-bridge.

In the example below the first two lines refer to the attached-detached couple and the second two lines refer to the detached-detached couple. The last line gives the position of the center of mass of the selected couple.

If[Random[] < 0.32,{a1 = m1 or + m2 (or + Cos[α Degree]/2),

a2 = or + 16.5 Cos[(110 Random[] + 40) Degree]/2},

{a1 = or + 16.5 Cos[(110 Random[] + 40) Degree]/2,

a2 = or + 16.5 Cos[(110 Random[] + 40) Degree]/2}];

a12 = N[(a1 + a2)/2];

The sequence above is repeated three times, one for each couple of the crone.

The position of the center of mass of the whole crone is:

d12 = (a12 + b12 + c12)/3
(13)

In the example below the angle, α, tilts from 140° to 104° with respect to the filament axis. The whole program is:

Aai = Table[{n,

Or = n d;

If[Random[] < 0.32,{a1 = m1 or + m2 (or + Cos[]/2),

a2 = or + N[16.5 Cos[(110 Random[] + 40) Degree]/2]},

{a1 = or + N[16.5 Cos[(110 Random[] + 40) Degree]/2],

a2 = or + N[16.5 Cos[(110 Random[] + 40) Degree]/2]}];

a12 = N[(a1 + a2)/2];

If[Random[] < 0.32,{b1 = m1 or + m2 (or + Cos[140 Degree]/2),

b2 = or + N[16.5 Cos[(110 Random[] + 40) Degree]/2]},

{b1 = or + N[16.5 Cos[(110 Random[] + 40) Degree]/2],

b2 = or + N[16.5 Cos[(110 Random[]+ 40) Degree]/2]}];

b12 = N[(b1 + b2)/2];

If[Random[] < 0.32,{c1 = m1 or + m2 (or + Cos[140 Degree]/2),

c2 = or + N[16.5 Cos[(110 Random[] + 40) Degree]/2]},

{c1 = or + N[16.5 Cos[(110 Random[] + 40) Degree]/2],

c2 = or + N[16.5 Cos[(110 Random[] + 40) Degree]/2]}];

c12 = N[(c1 + c2)/2];

d12 = N[(a12+b12 + c12)/3];d12},{n,0,48}];

48 d-Apply[Plus, aai[[Range[49],2]]]/49

This program gives the position of the center of mass of one array, cm_1_. Since the two arrays are not symmetric the program is repeated once more, this gives the position of the center of mass of the second array, cm_2_. The program is performed a first time with the value of the angle α at the beginning of the second phase and a second time with the value of the angle α at the end of the second phase. The difference between the sums of the position of the centers of mass at the beginning and at the end of the second phase:

(bcm_1_ + bcm_2_) − (ecm_1_ + ecm_2_)
(14)

is equivalent to the difference between the interference distances.

The difference between the sums of the positions of the center of mass equals the stroke size only when the head domains are fully attached and their tilting angles are all equal.

The programs were performed with Mathematica 4.
